# The Childhood-Onset Neurodegeneration with Cerebellar Atrophy (CONDCA) Disease Caused by *AGTPBP1* Gene Mutations: The Purkinje Cell Degeneration Mouse as an Animal Model for the Study of this Human Disease

**DOI:** 10.3390/biomedicines9091157

**Published:** 2021-09-04

**Authors:** Fernando C. Baltanás, María T. Berciano, Eugenio Santos, Miguel Lafarga

**Affiliations:** 1 Lab.1, CIC-IBMCC, University of Salamanca-CSIC and CIBERONC, 37007 Salamanca, Spain; esantos@usal.es; 2 Department of Molecular Biology and Centro de Investigación Biomédica en Red sobre Enfermedades Neurodegenerativas (CIBERNED), University of Cantabria-IDIVAL, 39011 Santander, Spain; berciant@unican.es; 3 Department of Anatomy and Cell Biology and Centro de Investigación Biomédica en Red sobre Enfermedades Neurodegenerativas (CIBERNED), University of Cantabria-IDIVAL, 39011 Santander, Spain; lafargam@unican.es

**Keywords:** AGTPBP1, CCP1, CONDCA, neurodegeneration, NNA1, pcd

## Abstract

Recent reports have identified rare, biallelic damaging variants of the *AGTPBP1* gene that cause a novel and documented human disease known as childhood-onset neurodegeneration with cerebellar atrophy (CONDCA), linking loss of function of the AGTPBP1 protein to human neurodegenerative diseases. CONDCA patients exhibit progressive cognitive decline, ataxia, hypotonia or muscle weakness among other clinical features that may be fatal. Loss of AGTPBP1 in humans recapitulates the neurodegenerative course reported in a well-characterised murine animal model harbouring loss-of-function mutations in the *AGTPBP1* gene. In particular, in the Purkinje cell degeneration (*pcd*) mouse model, mutations in *AGTPBP1* lead to early cerebellar ataxia, which correlates with the massive loss of cerebellar Purkinje cells. In addition, neurodegeneration in the olfactory bulb, retina, thalamus and spinal cord were also reported. In addition to neurodegeneration, *pcd* mice show behavioural deficits such as cognitive decline. Here, we provide an overview of what is currently known about the structure and functional role of AGTPBP1 and discuss the various alterations in AGTPBP1 that cause neurodegeneration in the *pcd* mutant mouse and humans with CONDCA. The sequence of neuropathological events that occur in *pcd* mice and the mechanisms governing these neurodegenerative processes are also reported. Finally, we describe the therapeutic strategies that were applied in *pcd* mice and focus on the potential usefulness of *pcd* mice as a promising model for the development of new therapeutic strategies for clinical trials in humans, which may offer potential beneficial options for patients with *AGTPBP1* mutation-related CONDCA.

## 1. Introduction

Childhood-onset neurodegeneration with cerebellar atrophy (CONDCA; OMIM 618276) is a recently identified, rare and severe autosomal recessive disease that affects the central and peripheral nervous systems. Individuals present an early global developmental delay resulting in cognitive decline and motor performance alterations, among other clinical features [1,2,3]. The severity of the disease is variable, and CONDCA can even result in death during childhood. Whole exome sequencing studies on CONDCA patients have identified different damaging biallelic variants of the *AGTPBP1* gene [1,2,3,4], linking *AGTPBP1* loss of function to human neurodegenerative diseases. Nevertheless, the deleterious effects of AGTPBP1 protein loss of function in animal models, especially in mouse models, have been known for some time [5,6,7,8,9,10,11].

Two decades ago, studies characterising genes involved in axonal regeneration in mice led to the identification of a 4-kb-long transcript hotspot. Due to its proposed role and nuclear localization, the gene encoding this transcript was named Nna1 (Nervous system Nuclear protein induced by Axotomy) [7]. Although the precise role of the protein encoded by the *AGTPBP1* gene remained elusive for several years, structural analysis of the AGTPBP1 protein revealed that it belonged to a new subfamily (M14D) of the M14 metallocarboxypeptidase family [12,13,14]. Subsequent functional analyses revealed that AGTPBP1, which acts as an enzyme, participates in post-translational modifications (PTMs) of tubulin. In particular, AGTPBP1 acts as a deglutamylase, catalysing the removal of polyglutamates at the C-terminal region of tubulin [15,16]. Other functions not directly related to tubulin processing, including maintenance of chromosomal stability and regulation of mitochondrial energy metabolism, were proposed for AGTPBP1 [17,18,19].

More than 40 years ago, the Purkinje cell degeneration (*pcd*) mouse model, which harbours a mutation that is autosomal recessive and displays distinct neurological deficits, causing profound ataxic behaviour, was established [5]. After extensive studies, the mutant gene responsible for the pcd mutation was mapped to mouse chromosome 13 and identified as the *AGTPBP1* gene [10].

The pcd mutation causes selective postnatal degeneration of certain neuronal populations, including the mitral cells (MCs) in the olfactory bulb (OB) [20], photoreceptors in the retina [6], certain subpopulations of thalamic neurons [21] and the Purkinje cells (PCs) in the cerebellum [10], the latter being responsible for the cerebellar ataxia of *pcd* mice. Moreover, a recent analysis has revealed that these animals also undergo peripheral nerve and spinal motor neuron degeneration [1]. Similarly, recent works have shown that excessive tubulin polyglutamylation in neurons, which results from AGTPBP1 dysfunction, alters the axonal transport of vesicles and appears to be the main mechanism of neurodegeneration [22,23,24]. In addition to ataxia, *pcd* mice also exhibit progressive cognitive impairments [9,25].

Interestingly, the well-characterised neurological deficits in *pcd* mice closely mimic the pathophysiology and clinical manifestations reported in CONDCA patients. Thus, the *pcd* mouse is an ideal animal model for investigating other probable but not yet characterised clinical alterations in patients with CONDCA. Moreover, potential therapeutic options for preventing, or at least attenuating, the neurodegenerative course in CONDCA patients could be assessed using this animal model.

Here, we summarise the current knowledge about the structure and function of the *AGTPBP1* gene in different cell types and tissues. We also review the variety of alterations in m*AGTPBP1* in *pcd* mice and their relationships with the pathological variants of the hA*GTPBP1* gene reported in CONDCA patients. Finally, we focus on the potential usefulness of the *pcd* mouse model as a suitable model for the clinical assessment of new pharmacological strategies and therapies that may offer possible treatment options for patients with *AGTPBP1* mutation-induced CONDCA.

## 2. The *AGTPBP1* Gene

### 2.1. Genomic Structure and Organisation

NNA1/AGTPBP1 contains a putative Walker A-box ATP/GTP binding motif (GXXGKS), which is highly conserved throughout evolution. According to the function and location of the protein encoded by this gene, it has also been called CCP1 (Cytosolic CarboxyPeptidase 1). Henceforth, we will use AGTPBP1 as the preferred term when referring to the gene or the protein.

**Figure 1:**
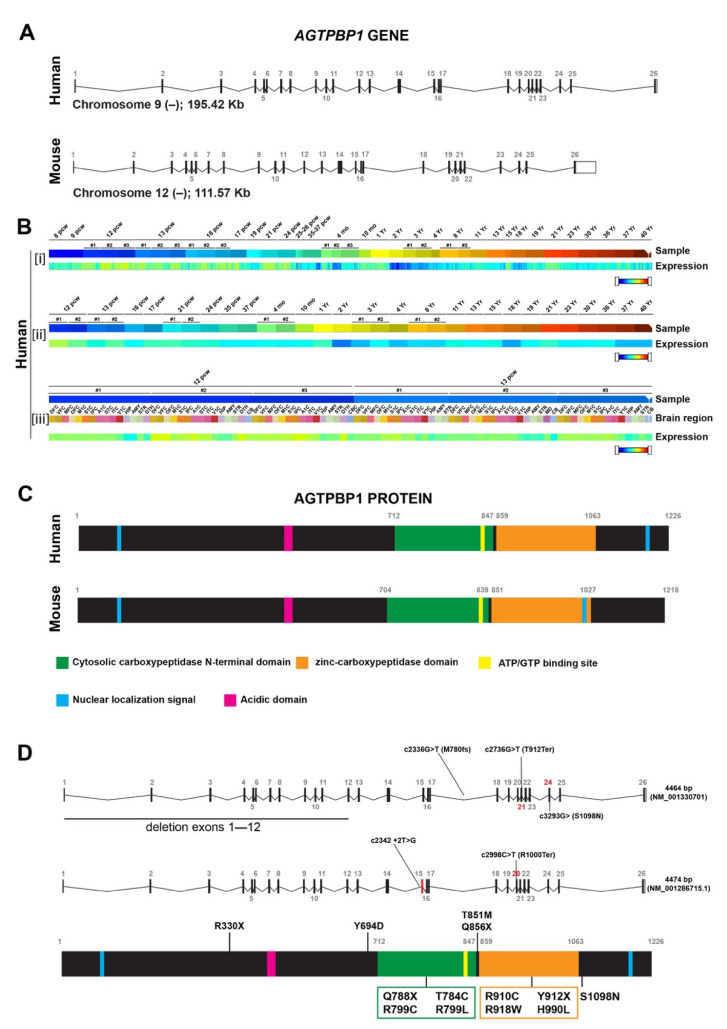
Genomic structure, organisation, expression and intron/exon distribution of AGTPBP1 in humans and in mice. (**A**) Genomic organisation of human *AGTPBP1* and mouse *AGTPBP1* loci. Figure assembled using current data from the Ensembl genome browser database (http://www.ensembl.org/index.html) (accessed on 10 July 2021). Chromosomal location, chromosome strand (+ or −) used for transcription, and the size (Kb) of the genomic stretches containing the *AGTPBP1* locus are also indicated for each species. Exons (solid vertical boxes) are numbered. The open box indicates the alternatively spliced region of the exon. (**B**) Schematic representation of [i] overall expression of the h*AGTPBP1* gene throughout development in all studied brain structures, [ii] the specific expression of the h*AGTPBP1* gene in the cerebellum throughout development, and [iii] region-specific hAGTPBP1 gene expression at 12–13 pcw. (**C**) Schematic representation of the primary structure of the human and mouse AGTPBP1 proteins. The structural domains and their relative positions are indicated. (**D**) Schematic representation of the human *AGTPBP1* gene structure and the encoded protein and the locations of variations found in patients with AGTPBP1 mutations. DFC: dorsolateral prefrontal cortex; VFC: ventrolateral prefrontal cortex; MFC: anterior (rostral) cingulate (medial prefrontal) cortex; OFC: orbital frontal cortex; M1C: primary motor cortex; S1C: primary somatosensory cortex; IPC: inferior parietal cortex; A1C: primary auditory cortex; STC: superior temporal cortex; ITC: inferolateral temporal cortex; V1C: primary visual cortex; HIP: hippocampus; AMY: amygdaloid complex; STR: striatum; DTH: dorsal thalamus; CB: cerebellum; CBC: cerebellar cortex. PWC: postconception weeks; MO: Months; Yr: Year.

The genomic regions occupied by the *AGTPBP1* locus vary widely between different organisms, but the intron/exon distribution of this gene is highly conserved. For example, the h*AGTPBP1* gene is located on chromosome 9 (Chr 9q21.33 position: 85,546,539–85,742,029; 26 exons), whereas the m*AGTPBP1* gene is located on chromosome 13 (Chr13 position: 59,445,742–59,585,227; 26 exons) (Figure 1A).

### 2.2. Expression Pattern

h*AGTPBP1* mRNA or protein is detectable in practically all human cells, tissues and organs tested. Of note, the mRNA and protein expression levels of h*AGTPBP1* differ significantly depending on the specific organ and tissue.

According to the consensus dataset of the Human Protein Atlas database obtained based on transcriptomic analyses of human tissue and organs, the mRNA expression of h*AGTPBP1* is the highest in bone marrow (https://www.proteinatlas.org/ENSG00000135049-AGTPBP1/tissue) (accessed on 10 July 2021) followed by many regions of the central nervous system, including the spinal cord, pons and medulla, corpus callosum, cerebellar cortex, hippocampal formation and olfactory region, among others. h*AGTPBP1* mRNA expression is generally higher in the brain than in all other non-brain tissues. For example, low mRNA expression levels are detected in the ovary, liver, stomach, small intestine, lung, adrenal gland, spleen, and thymus, among other tissues. Similar distribution patterns of m*AGTPBP1* mRNA were found in mouse tissues [7,12]. In brain regions, m*AGTPBP1* is expressed preferably in differentiating neurons rather than in proliferating precursors/progenitors. [7].

Using the BrainSpan Developmental Transcriptome database, which contains data related to human gene expression in 16 specific brain structures obtained using RNA sequencing and exon microarray analysis of 42 brain samples spanning pre- and post-natal development, both the temporal and regional specificity of h*AGTPBP1* gene expression in the brain can be examined (https://www.brainspan.org/rnaseq/searches?exact_match=false&search_term=%22NNA1%22&search_type=gene) (accessed on 10 July 2021). Regarding the temporal pattern of h*AGTPBP1* expression, overall higher h*AGTPBP1* gene expression is detected during embryonic development than during childhood and adulthood (Figure 1B, panel i), with the expression of the gene peaking at approximately 12–13 postconception weeks (pcw). In contrast, the lowest h*AGTPBP1* gene transcription is found in brain samples from children (2–8 years old) (Figure 1B, panel i). A very similar expression pattern is found in the cerebellum and cerebellar cortex throughout development (Figure 1B, panel ii). With regards to regional-specific expression, in the specific time windows in which the h*AGTPBP1* gene shows the highest transcription levels (~12–13 pcw), the highest h*AGTPBP1* gene expression levels are found in the prefrontal and frontal cortices and the dorsal thalamus, whereas the lowest levels are detected in the hippocampus, cerebellum and striatum (Figure 1B, panel iii).

## 3. The AGTPBP1 Protein

### 3.1. Modular Domain Structure

The h*AGTPBP1* gene encodes a 1226-amino acids (aa) protein (https://www.uniprot.org/uniprot/Q9UPW5) (accessed on 10 July 2021) containing a P-loop ATP/GTP-binding motif and a nucleotide-binding site. The primary structure of the hAGTPBP1 protein is a sequential, linearly organised modular configuration featuring conserved distribution of two well-defined domains: the cytosolic carboxypeptidase N-terminal domain (aa 712–847), which is highly conserved among M14D subfamily members [12,26], and the catalytic zinc-carboxypeptidase domain (aa 859–1063; Figure 1C). The ATP/GTP binding site is at aa 820–825, and an active catalytic site is found at position 970. Using cNLS mapper software (http://nls-mapper.iab.keio.ac.jp/cgi-bin/NLS_Mapper_form.cgi) (accessed on 10 July 2021), two bipartite nuclear localisation signals (NLSs) were predicted: a signal at the N-terminal region (aa 120–149) and a signal situated at the C-terminus (aa 1139–1165). In addition, other key residues directly related to the function of the protein were identified, including zinc-binding sites in the hAGTPBP1 protein (residues 920, 923 and 1017) [12]. Importantly, the hAGTPBP1 protein is highly evolutionarily conserved, sharing ~72.5% identity with the AGTPBP1 protein in D. melanogaster and D. rerio and 87.2% identity with the AGTPBP1 protein in mice, supporting the notion that this gene, especially the catalytic domains, is highly conserved in metazoans [26,27,28]. The mA*GTPBP1* gene encodes a 1218-aa protein (https://www.uniprot.org/uniprot/Q641K1) (accessed on 10 July 2021) that also contains a cytosolic carboxypeptidase N-terminal domain (aa 704–839; Figure 1C) and a zinc-carboxypeptidase domain (aa 851–1027; Figure 1C). The ATP/GTP binding site is at aa 810–817, and the active site is located at residue 962. Whereas mutation of the ATP/GTP binding site has no effect in vivo, preservation of a functional zinc-binding domain is essential for neuronal survival [14,29,30]. Zinc-binding sites in the mAGTPBP1 protein are found at residues 912, 915 and 1009. The NLSs are positioned at both the N-terminal domain (aa 144–151) and C-terminal domain (aa 996–1016) [9].

### 3.2. Expression Pattern

hAGTPBP1 protein expression level data have revealed that the protein is expressed at the highest level in the testis (https://www.proteinatlas.org/ENSG00000135049-AGTPBP1/tissue) (accessed on 10 July 2021). Moderate protein expression levels are also observed in human brain regions, such as the cerebral cortex and cerebellum, and other organs, including the lung, stomach, kidney, pancreas, and muscle.

At the cellular level, the AGTPBP1 protein is detected both in the cytoplasm, mainly in vesicles and mitochondria, and in the nucleus, particularly in the nucleolus (https://www.proteinatlas.org/ENSG00000135049-AGTPBP1/cell) (accessed on 10 July 2021), as reported for mAGTPBP1 [7,18,26].

### 3.3. Role of the AGTPBP1 Protein

Microtubules are major components of the cytoskeleton composed of α- and β-tubulin heterodimer subunits that polymerise to form tubular and polar structures [31]. Both tubulin subunits are subject to certain PTMs, including the tyrosination/detyrosination and polyglutamylation of α-tubulin [32]. The tyrosination/detyrosination cycle involves the reversible removal and re-addition of a tyrosine residue at the C-terminus of α-tubulin. Two enzymes participate in this cycle: tubulin tyrosine ligase (TTL) and tubulin carboxypeptidase (TubCP). The latter cleaves the C-terminal tyrosine residue of α-tubulin, resulting in Glu-tubulin. The tyrosine residue can be re-added by TTL, forming Tyr-tubulin again. Another variant of non-tyrosinable α-tubulin (Δ2-tubulin) that lacks the Glu C-terminal residue was defined.

**Figure 2:**
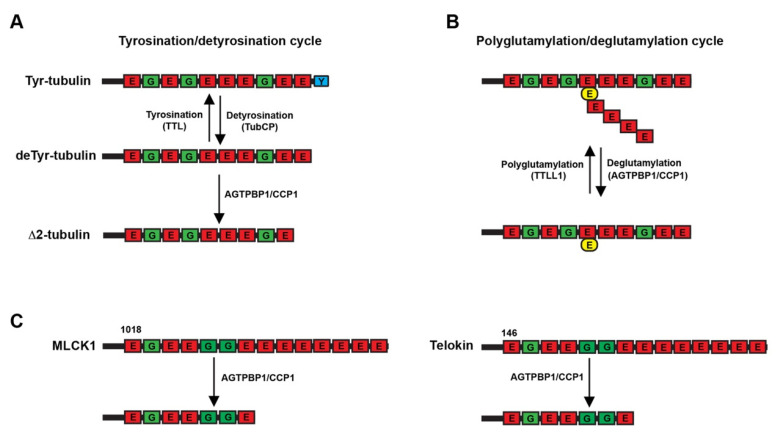
AGTPBP1/CCP1-mediated tubulin posttranslational modifications. (**A**,**B**) Schematic representation of the two AGTPBP1/CCP1-mediated tubulin posttranslational modifications. (**C**) Schematic representation of the deglutamylation of MLCK1 (left) and telokin (right) catalysed by AGTPBP1/CCP1. The numbers indicate the amino acid residues. MLCK1: Myosin light chain kinase 1. TTL: Tubulin Tyrosine Ligase; TTLL1: Tubulin Tyrosine Ligase-Like 1; TubCP: Tubulin Carboxypeptidase. Scheme is a modification of [15].

Several years ago, the enzymes catalysing tubulin PTMs were fully characterised [33]. Cytosolic carboxypeptidase (CCP) subfamily comprises six members (CCP1-CCP6), [12,13]. CCP enzymes process amino acid residues from the C-terminus [15,34] and are also involved in reversing polyglutamylation catalysed by polyglutamylases from the tubulin-tyrosine ligase-like family and in removing glutamate residues from the C-terminus, thus transforming α-tubulin into non-tyrosinable Δ2-tubulin [15] (Figure 2A,B). In particular, CCP1, encoded by the *AGTPBP1* gene, acts as a tubulin deglutamylase, generating Δ2-tubulin from Glu-tubulin, and also counteracts the tubulin–tyrosine ligase-like mediated reaction and then shortens the long glutamate chains generated by polyglutamylases [15,16,35]. In addition, myosin light chain kinase and telokin proteins were also identified as substrates for CCP1 [15] (Figure 2C).

Although AGTPBP1 contains two NLSs [9] and is expressed in the nucleus [7], its contribution to nuclear physiology remains uncertain; however, it was suggested that this protein may be involved in chromatin remodelling [7].

## 4. *AGTPBP1* Mutation-Related Childhood-Onset Neurodegeneration with Cerebellar Atrophy (CONDCA)

Shashi and colleagues (2018) identified biallelic variants in the *AGTPBP1* gene in patients suffering from an infantile-onset neurodevelopmental disorder known as childhood-onset neurodegeneration with cerebellar atrophy (CONDCA) [1]. At the time this review was written, a total of 18 individuals from 15 different families were reported to carry damaging CONDCA-associated, biallelic, loss-of-function variants of the *AGTPBP1* gene (Table 1). The affected individuals, most of them unrelated, were born from both consanguineously related and unrelated parents, and the disease appeared to equally affect both males and females (Table 2).

Complete exome sequencing analysis of CONDCA patient samples revealed rare allelic variants of the *AGTPBP1* gene, including (i) missense mutations predicted to result in single aa substitutions; (ii) a canonical splice-site change causing disturbance of the open reading frame; (iii) de novo heterozygous stop-gain variants predicted to generate a premature stop codon; (iv) an intronic splice-site change predicted to lead to the in-frame absence of 29 highly conserved aa; and (v) a 12-exon-long genomic deletion (Table 1; Figure 1D) [1,2,3,4]. The majority of these variants affect the highly conserved N-terminal regions and zinc-carboxypeptidase protein motifs (Figure 1D). As a consequence, the function of the AGTPBP1 protein is abrogated due to a reduction in mRNA transcription, nonsense-mediated mRNA decay, early protein truncation and protein misfolding into a structure that is highly susceptible to proteasome-dependent proteolysis [1].

**Figure 3:**
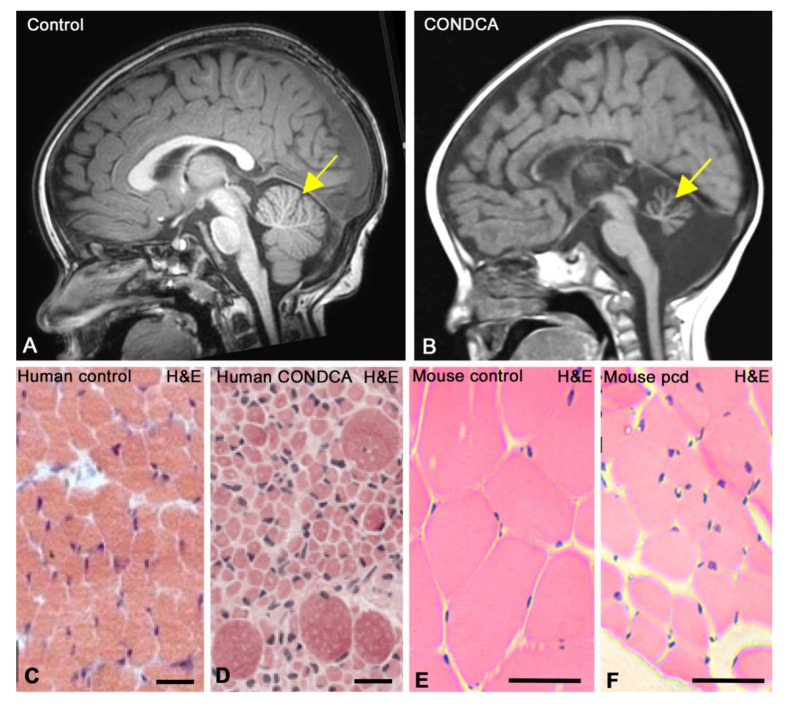
Cranial magnetic resonance imaging (MRI) of a control (**A**) and a CONDCA patient (**B**). Whereas the 20-month-old female healthy control patient (**A**) shows a typical well-developed cerebellum (yellow arrow), severe cerebellar atrophy (yellow arrow) is observed in the 24-month-old female CONDCA patient (**B**). (**A**) Courtesy of Dr. Ana Canga, “Hospital Universitario Marqués de Valdecilla”, Santander (Spain). (**B**) Adapted from [3]. Copyright 2019 American Journal of Medical Genetics. (**C**,**D**) Haematoxylin-eosin (H&E)-stained skeletal muscle tissue biopsies at 7 months of age from healthy control (**C**) and CONDCA patients (**D**). Note the fibre atrophy with a few interspersed hypertrophic fibres in the muscle tissue of the patient. Scale bars: 50 µm. Adapted with permission from [1]. Copyright 2018 The EMBO Journal. (**E**,**F**) Haematoxylin-eosin (**E**,**H**)-stained skeletal muscle tissue cross-sections from control (**E**) and *pcd* (**F**) mice. Note the muscle atrophy and the notable reduction in muscle fibre size in the *pcd* mouse. Scale bars: 30 µm. Adapted with permission from [37]. Copyright 2018 Journal of Tissue Engineering and Regenerative Medicine.

The clinical findings of patients with AGTPBP1 mutations are summarised in Table 2. Overall, the patients display early-onset developmental delays (between birth and 20 months of age) with a progressive degenerative course, mainly characterised by hypotonia and generalised muscle weakness, frequently causing tetraparesis. Brain MRI revealed detected cerebellar atrophy with respect to non-affected, control individuals (Figure 3A,B). Other brain alterations, such as microcephaly or dysplastic corpus callosum, were also frequently detected. Alterations in tendon reflexes were observed in almost all individuals. Muscle atrophy was found in half of the patients (Figure 3C,D), and other regularly detected clinical manifestations included feeding problems, eye movement abnormalities, respiratory insufficiency, spasticity, tongue fasciculations and dystonia. Other clinical features, such as bilateral hearing loss and hand tremors, were sporadically detected.

Electrophysiological recordings revealed motor neuropathy affecting the lower limbs and arms. In particular, electromyography studies have detected signs of denervation causing muscle atrophy in proximal and distal muscles, including the tibialis anterior and posterior deltoid, suggesting degeneration of both peripheral nerve motor fibres and spinal cord α-motor neurons. In contrast, sensory nerve action potentials seemed to be unaffected, suggesting that the neuropathy was mainly of motor origin. Progressive advancement of the neurological disorders resulted in the death of 7 patients out of the 18 individuals carrying damaging variants of the *AGTPBP1* gene.

It is relevant to mention that in a very recently published paper, two members of a consanguineous family harbouring a novel homozygous variant (c.3293G>A) at the 3′ end of the *AGTPBP1* gene (Figure 1D) showed no signs of cerebellar atrophy [36]. Further analysis is needed to examine whether only AGTPBP1 mutations affecting the catalytic domains of the protein are directly associated with cerebellar atrophy.

At the cellular level, deleterious accumulation of polyglutamylated tubulin was detected in biopsies taken from the quadriceps muscles of CONDCA patients with AGTPBP1 mutations [1]. Additionally, this hyperglutamylation is directly related to neurodegeneration in mice and humans, most likely due to deficiency in microtubule-based axonal transport [38].

Interestingly, a recent study has identified the *AGTPBP1* gene as being the most significant gene coexpressed with the amyotrophic lateral sclerosis (ALS)-linked gene C90RF72 and revealed a positive correlation between the expression of their respective mRNAs [39]. These findings suggest that AGTPBP1 is an interacting partner of C90rf72 that contributes to the regulation of important neuronal functions [39]. This raises questions regarding the potential role of AGTPBP1 in other human neurological disorders.

## 5. The *pcd* Mouse as an Animal Model for Studying *AGTPBP1* Mutation-Related CONDCA

More than 40 years ago, a spontaneous recessive mutation that causes early cerebellar ataxia and is associated with rapid degeneration of cerebellar PCs was identified [5]. This mutation was subsequently called pcd [5]. To date, damaging mutations in AGTPBP1, including spontaneous mutant variants, chemically induced variants, transgenic alleles and conditional knockout, were reported (Table 3). The most severe pathological alleles are pcd^1J^, pcd^3J^, pcd^5J^ and pcd^JWG^ [9]. The pcd^2J^ allele is a less severe hypomorphic variant that causes a mild phenotype and the development of ataxia much later than other pcd mutants but not thalamic degeneration (Table 3). A recently generated AGTPBP1 KO mouse model in which exons 21–22 are deleted [40], exhibits similar pathological features as those reported for pcd1J, pcd^3J^ or pcd^5J^ (Table 3).

Loss of function of the *AGTPBP1* gene was subsequently identified as being responsible for the *pcd* phenotype [7,10]. *pcd* mice have a smaller body and lower body weight than their wild-type counterparts [35,40]. In addition to PC degeneration, the pcd mutation leads to postnatal degeneration of other distinct neuronal populations, including MCs in the OB [20], photoreceptors in the retina [6], a certain subpopulation of thalamic neurons [21] and peripheral nerve and spinal motor neurons [1].

Interestingly, the time course of degeneration of affected neuronal populations in *pcd* mice markedly differs among them, making this animal model highly appropriate for studying different neuronal degenerative processes caused by the same mutation. Degeneration of photoreceptors is slow and takes approximately one year [6,41] whereas the death of thalamic neurons and MCs takes up to 6 and 4 months, respectively [20,21]. In contrast, degeneration of PCs occurs extremely quickly, is severe and occurs between 3 and 4 weeks of age. In fact, almost all PCs are eliminated at a well-defined time point, approximately by 1 month after birth. To date, no clear findings supporting marked variations in these neurodegenerative processes exist. Since most *pcd* mice survive, secondary neuronal death and remodelling of neuronal networks, as consequences of primary neurodegeneration, were reported in the affected regions.

Interestingly, cerebellar atrophy is one of the earliest signs of the disease manifested in CONDCA patients, which resembles the early PC degeneration and the cerebellar atrophy showed by *pcd* mice. Nevertheless, although the correspondence of other clinical findings between CONDCA patients and *pcd* mice is established (Table 4) it still remains unknown if the developmental course of the pathologies is comparable in both groups. With the currently available data, the assumption of similar developmental phases of the disease between CONDCA patients and *pcd* mice cannot be yet established.

In summary, the *pcd* mouse is a suitable animal model for studying ataxia and cerebellar atrophy with genetic, clinical and histopathological characteristics similar to those of human CONDCA patients (Table 4). The following sections describe in detail the neuropathological signs and mechanisms of neurological dysfunction in *pcd* mice.

### 5.1. Degeneration in the Cerebellum

One of the main phenotypic hallmarks of the pcd mutation in mice is early-onset cerebellar atrophy, which is mainly associated with drastic and premature primary degeneration of PCs [19,40], starting in the vermis and progressively advancing to the cerebellar hemispheres [42]. As degeneration proceeds, other cerebellar neuronal populations, such as granule cells and neurons of the inferior olivary complex and deep cerebellar nuclei (DCN), subsequently degenerate. However, this secondary degeneration process is much slower, taking approximately one year, probably as a consequence of primary PC death [9]. Consequently, there is an additional thinning of the molecular and granule cell layers with the subsequent worsening of the cerebellar atrophy [43,44].

At the molecular level, analysis of the transcriptional signature in the cerebella of *pcd* mice has shown that the vast majority of genes with altered transcriptional levels are related to functional categories such as cell death, developmental disorders, survival and glial responses [45,46].

#### 5.1.1. Degeneration of Purkinje Cells

**Figure 4:**
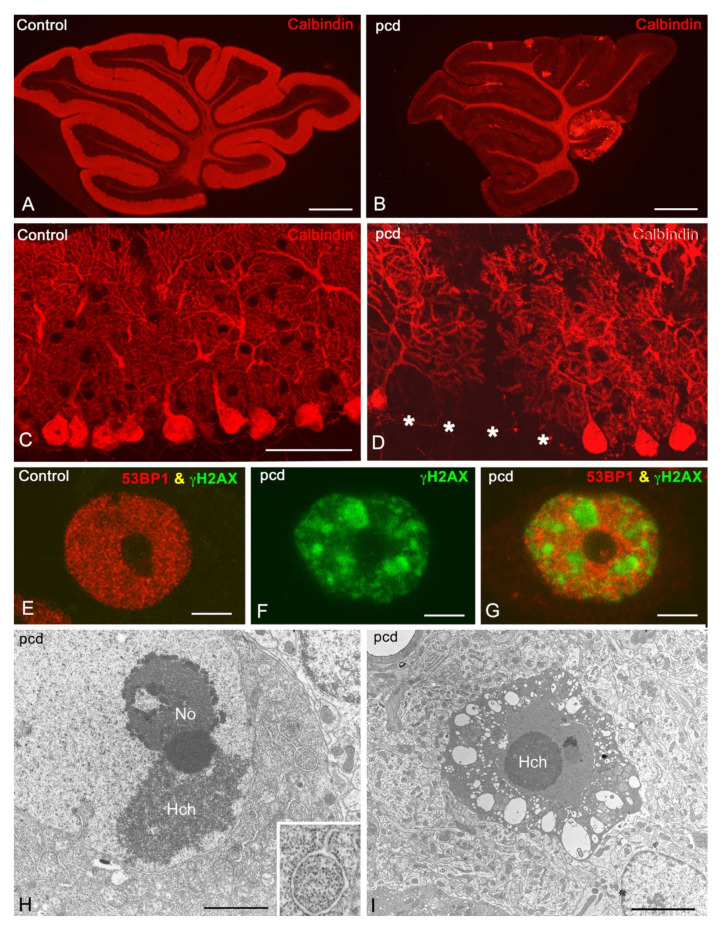
Degenerative features of the Purkinje cells in the *pcd* mice. (**A**,**B**) Representative confocal microscopy images of sagittal sections of the vermis of P30 control (**A**) and P30 (**B**) *pcd* mutant mice immunolabelled for calbindin D-28k. Note that in the *pcd* mouse there was a dramatic reduction in calbindin immunostaining in the molecular and PC layers resulting from the massive loss of PCs at P30 and that only PCs located in lobule X remained (arrow in B). Scale bars: 1 mm. (**C**,**D**) High magnification of calbindin D-28K immunolabelling of PC perikarya and their dendritic trees in control (**C**) and *pcd* mice (**D**) at P20. Note the loss of PCs (white asterisks) in the *pcd* mouse. Scale bar: 100 µm. (**E–G**) Confocal microscopy images of PC nuclei from control (**E**) and *pcd* mice (**F**,**G**) at P20 double immunolabelled for the modified histone γH2AX (green), a marker of DNA double-strand breaks at sites of DNA damage, and p53-binding protein 1 (P53BP1, red), a key DNA repair factor. (**E**) Note the absence of γH2AX labelling and the typical diffuse nucleoplasmic distribution of 53BP1, in the control PC nucleus, excluding the nucleolus. (**F**,**G**) In contrast, the nucleus of the *pcd* mouse shows prominent nuclear foci of DNA damage immunostained for γH2AX (**F**). Although the DNA repair factor 53BP1 was expressed in the nucleoplasm, it was not concentrated in γH2AX-positive nuclear foci of DNA lesions (**G**), indicating defective DNA repair. Scale bars: 5 μm. (**H**) Electron microscopy image of PCs from *pcd* mice at P20. Free polyribosomes were replaced by densely packed monoribosomes. Cytoplasmic portions containing monoribosomes appear sequestered in autophagic vacuoles bound by isolated RE cisternae (insert). Scale bar: 1 μm. (**I**) Electron microscopy image of mutant apoptotic PC Scale bars: 5 μm. (**A**,**B**) Adapted with permission from [17]. Copyright 2011 The Journal of Biological Chemistry. (**C**,**D**,**H**) Adapted with permission from [48]. Copyright 2011 Brain Pathology. (**E**–**G**) Adapted with permission from [49]. Copyright 2019 Neurobiology of Disease.

One of the phenotypic hallmarks of the pcd mutation in mice is early-onset cerebellar atrophy, which is mainly associated with drastic primary degeneration of PCs. Between two and four weeks of age, PCs rapidly degenerate, with only a few PCs remaining in lobule X of the cerebellar vermis, which is preserved for a few additional weeks (Figure 4A,B) [10,17,19,47]. The degeneration of a massive number of PCs in *pcd* mice sequentially involves an initial “preneurodegenerative” stage, from postnatal day (P) 15 to P20, during which both cytoplasmic and nuclear alterations occur [17,25,48,49], followed by a degenerative stage (P25–45), in which all cerebellar PCs degenerate (Figure 4C,D) [17,48], leading to an alteration in cerebellar-related motor performance. Interestingly, heterozygous *pcd* mice show a significant reduction in the number of PCs at P300, an observation that supports the idea that heterozygosity of the AGTPBP1 mutation may influence the ageing process, causing moderate PC degeneration [50].

At the early stages of PC degeneration, alterations in the cytoarchitecture of the PCs, including disrupted dendrites, soma and axons, are already noticeable [40,46]. Ultrastructural analysis of axonal torpedoes has revealed organelle accumulation and cytoplasmic densification but the preservation of the myelin sheath [46]. This finding suggests that degeneration of axons is the primary defect and rules out the possibility that PCs degenerate following demyelination, as observed in the cerebral cortex in AGTPBP1-deficient mice [22]. AGTPBP1 loss of function affects dendritic tree development and architecture; however, these alterations do not seem to be directly involved in PC death [19,25,40].

Another pre-degenerative characteristic of PCs is a reduction in perikaryal size [25]. Intriguingly, one of the earliest cytoplasmic morphological features of pre-degenerative PCs is the accumulation of free polyribosomes [19,47,51]. Polyribosome accumulation correlates with endoplasmic reticulum (ER) stress in the PCs of *pcd* mice. In the initial stages of PC degeneration, the stacks of ER cisterns tend to disappear, and PCs show a prominent mass of densely packed free polyribosomes at the basal pole of PC somas. As degeneration proceeds, polyribosomes are disassembled into free monoribosomes [48]. A fraction of these free monoribosomes is sequestered in autophagic vacuoles for lysosomal degradation through a process termed ribophagy (Figure 4H) [48].

At the molecular level, upregulation of the expression of ER stress-related substrates and the unfolded protein response accompanied by downregulation of the expression of initiation factors for translation were detected in the mutant PCs [40,51,52]. Accumulation of hyperglutamylated tubulin in AGTPBP1-deficient PCs directly correlates with ER stress [51]. Other cytoplasmic alterations, such as the presence of abnormal mitochondria and reduced mitochondrial complex 1 activity, were also observed in mutant PCs [18,53].

The AGTPBP1 gene contains an NLS (Figure 1B) and encodes nuclear and cytoplasmic proteins [7]. However, little is known about its potential role in the neuronal nucleus. We performed an extensive analysis of the effects of AGTPBP1-deficiency in the PC’s nuclear compartments involved in RNA transcription and processing and DNA damage repair, evaluating the impact of their dysfunction on neuronal homeostasis and survival [17,48,49]. During the pre-degenerative stage, in PCs, there is a progressive large-scale reorganisation of chromatin into large, transcriptionally silent, heterochromatin domains associated with the accumulation of DNA damage, which is one of the first signs of the PC pre-neurodegenerative stage of in *pcd* mice [17].

To avoid the detrimental effects of DSBs, neurons exhibit a strong DNA repair response. However, growing evidence has indicated that defective DNA repair is the basis for brain ageing and several degenerative disorders [54]. In this context, a progressive accumulation of unrepaired DNA damage was detected in the PCs of *pcd* mice (Figure 4E–G) [17,48]. Accordingly, defective DNA repair was implicated in the pathogenesis of several ataxias with a PC degeneration phenotype [55,56,57,58,59,60].

In addition to DNA damage and epigenetic changes in chromatin conformation, other nuclear compartments are affected in the mutant PCs. In particular, the disassembly of the Cajal bodies [61] and nucleolar disruption are directly correlated with the activation of nucleolar stress and defective ribosome biogenesis [48,49]. Nucleolar stress has been associated with several neurodegenerative human disorders, including Alzheimer’s and Parkinson’s diseases, ALS and spinal muscular atrophy, among others [62,63,64,65,66,67,68]. Moreover, a reduction in both nucleolar size and ribosome biogenesis occurs during ageing and is a key risk factor related to the onset of neurodegenerative disorders [54,69].

Consistent with all defined cytoplasmic and nuclear alterations, dysfunctional PCs in *pcd* mice ultimately activate the caspase-mediated apoptotic pathway (Figure 4I), including by upregulating the expression of neuronal apoptosis facilitators such as Bim3 and Bcl2l11 [9,17,18,19,48,51,52,70]. In summary, some types of nuclear rearrangement and pathological alterations observed in PCs harbouring the pcd mutation resemble the cellular alterations described in certain human neurodegenerative diseases [66,71,72].

#### 5.1.2. Alterations in Other Neuronal Types in the Cerebella of *pcd* Mice

In the cerebella of *pcd* mice, late secondary death of granule cells and neurons in the DCN and the inferior olivary complex, which is likely due to PC degeneration, occurs [9,43,73]. Remarkably, granule cells express the AGTPBP1 gene [7,10]. However, a significant reduction in granule cell number is only detected at 6 months of age, which progresses throughout the lifespan of the animal [9].

The loss of presynaptic afferents from PCs to the DCN results in decreased neuron survival (~30%) at advanced stages of neurodegeneration [44,74]. As a consequence of the abrogation of its major cortical target, the inferior olivary complex becomes atrophied subsequent to partial denervation in *pcd* mice [40,43,75]. In particular, the number of neurons in the inferior olivary complex is reduced by half at 10 months of age, which then causes a reduction in the number of climbing fibres that reach the cerebellar cortex [75].

Finally, the disappearance of PCs in *pcd* mice stimulates severe gliosis [40,46] and strikingly, oligodendrocytes and their precursors are markedly affected in the cerebellar cortex in *pcd* mice [46].

#### 5.1.3. Reorganisation of Cerebellar Circuitry in *pcd* Mice after Purkinje Cell Loss

The compensatory mechanisms in the cerebellar circuitry following PC loss in *pcd* mice were examined [76]. Electrophysiological studies have revealed that despite the absence of PC-mediated tonic inhibition in the vestibular nucleus (VN) in *pcd* mice, spontaneous activity is not greater in AGTPBP1-deficient neurons [76]. In addition, abrogation of PC input did not underlie disinhibition in neurons from the VN in *pcd* mice [76]. The influence of PC input loss in VN neurons in regulating muscle contraction through the vestibulospinal pathway was also assessed. The response phase is slightly modified in the *pcd* mice in comparison with control littermates. This effect may be partially involved in motor impairment [76].

The impact of AGTPBP1 deficiency on cerebellar neurotransmission was also evaluated. Abrogation of inputs originating from PCs reduces GABAergic inhibitory innervation and decreases the density of GABAA receptors in the DCN and VN [74,76]. In contrast, the glycinergic system was promoted in the DCN in *pcd* mutant mice [74]. On the other hand, an increase in the number of glutamatergic synapses in both the DCN and the VN was detected, most likely due to enhanced mossy fibre innervation of the DCN and a secondary effect of reduced GABAergic-mediated inhibition in DCN neurons [74].

PCs and granule cells are specific targets for serotonergic neurons projecting from raphe nuclei and other brain areas. The serotonergic centrifugal system in the cerebella of *pcd* mice becomes dysfunctional once PCs are completely depleted [77]. In particular, *pcd* mice showed a higher 5-HT-IR fibre density [78] and a reduction in the 5-HIAA/5-HT ratio [77]. Enhanced synthesis of 5-HT transporters and receptors was also detected in both the cerebellar cortex and DCN [77,79]. Together, these data point to a reduction in serotonergic modulation, indicating a decrease in serotonergic turnover in the *pcd* cerebella [77].

Noradrenergic axon terminals from the locus coeruleus that reach the cerebellar cortex are preserved in *pcd* mice despite the absence of PCs, the targets of noradrenergic projections [80,81]. In addition, an increase in the density of norepinephrine fibres, most likely due to a reduction in cerebellar mass in *pcd* mice, was also detected [81]. Likewise, a moderate increase in the levels of noradrenergic transporters and adrenergic receptors upon PC loss was observed [82]. Regarding the dopaminergic system, PC loss induces an increase in the levels of dopamine transporters in the DCN but a significant reduction in these levels in the molecular layer of the cerebellar cortex [83].

#### 5.1.4. Alterations in Cerebellar-Dependent Tasks

A large battery of motor tests were used to evaluate the degree of cerebellar atrophy and the consequent impairment of locomotor coordination, as well as progressive weakness of musculature and cognitive decline in AGTPBP1-depleted mice.

Analysis of ataxic gait has revealed irregularly spaced and shorter steps in 4-week-old mutants, with this deficit becoming worse as cerebellar degeneration proceeds [14,40]. Severe early impairments in motor performance, in the rotarod test, which progressively deteriorates in parallel with PC death, were observed in *pcd* mice [14,17,19,25,40,84,85]. It was demonstrated that in the treadmill motor assessment, *pcd* mice have decreased body movement coordination [84]. Similarly, front-hind interlimb and whole-body coordination deficits were characterised using LocoMouse [86].

The grip strength and wire hang tests have revealed that *pcd* mice experience muscle weakness [35,40]. In addition, the balance beam test has revealed that 4-week-old AGTPBP1 KO mice fall significantly more frequently than their wild-type counterparts [40].

Delayed eye-blinking conditioning appears to be severely affected and altered cerebellum-dependent learning is altered in adult *pcd* mice [87], whereas trace eyeblink conditioning is unimpaired, suggesting that the cerebellum plays an indispensable role in the neuronal circuitry regulating this response [88]. The effects of cerebellar dysfunction on spatial learning in adult *pcd* mice were determined using the Morris water maze test [89,90,91]. The novel object recognition test has revealed that long-term memory in *pcd* mice is affected in the late stages of PC degeneration, and the results of the social preference test have suggested that PC loss in *pcd* mice affects social interaction [25]. Likewise, the results of the forced swimming test have suggested that *pcd* mice exhibit depressive-like behaviour [91].

### 5.2. Degeneration in the Olfactory Bulb

#### 5.2.1. Degeneration of Mitral Cells

The OB is considerably smaller in *pcd* mice than in control animals, mainly due to the loss of MCs, the principal relay neurons in the olfactory pathway [5]. The loss of MCs is accompanied by reductions in the size of glomeruli and the thickness of the external plexiform layer. Other bulbar layers or neural elements are apparently unaffected by the loss of AGTPBP1 [8].

Degeneration of MCs occurs later and more slowly than that of PCs, taking place from P60 to P90 days [8,20]. While there is an extensive amount of data on the mechanisms underlying PC death, there is limited information regarding the mechanisms involved in MC degeneration. Similar to that of mutant PCs, degeneration of MCs is associated with ER stress, transcriptional repression, DNA damage and disruption of nucleoli and Cajal bodies, which ultimately cause apoptosis [20]. As in the cerebellum, tubulin hyperglutamylation in the OB was suggested to be a determinant of MC death in *pcd* mice [15,16].

MC degeneration induces reactive glial activation of astrocytes and microglia in the OB. However, this response is milder than that detected in the cerebellum [46]. Curiously, bulbar oligodendrocytes are not affected in *pcd* mice [46]. Differential glial responses observed in the cerebellum and the OB seem to correlate with the degree of neurodegeneration in each brain region and physiological AGTPBP1 expression levels [46].

#### 5.2.2. Reorganisation of Synaptic Circuitry after Mitral Cell Loss

MCs establish reciprocal dendrodendritic synapses with bulbar granule cells. Although the pcd mutation does not compromise the viability of granule cells, MC degeneration prevents afferent inputs from contacting granule cells. Some granule cells establish new reciprocal dendrodendritic synapses with unaffected tufted cells [92]. However, it should be noted that mutant granule cells have an effect on the dendritic tree, including shortening dendrites and reducing the number of spines [93]. In contrast, afferent inputs reaching the OB from olfactory receptor cells are slightly affected by the loss of MCs [8].

MCs send axonal efferent inputs to the lateral olfactory tract. A general decrease in the thickness of the olfactory tract was found in *pcd* mice [92], supporting the notion that the number of synapses declines upon MC degeneration. In addition, the diameter of terminal boutons increases, as does the number of multiple synaptic contacts, in *pcd* mutants, suggesting further compensatory mechanisms for the loss of MC presynaptic terminals [92].

Centrifugal afferences from secondary olfactory structures to the OB upon MC loss were also examined. Strengthening of the centrifugal input to the OB from the anterior olfactory nucleus after MC loss was detected in *pcd* mice and is accompanied by complete loss of bilaterality in olfactory connections due to degeneration of the anterior commissure [94]. These results point to a dramatic reorganisation of this essential olfactory circuit between the anterior olfactory nucleus and the OB upon MC degeneration.

Regarding the dopaminergic system in the OB, autoradiography studies have shown that dopamine receptor and transporter levels are not affected by AGTPBP1 loss of function [83]. Accordingly, tyrosine hydroxylase activity and immunoreactivity in OB juxtaglomerular neurons are more preserved in *pcd* mutants after MC degeneration than in heterozygous littermates [95].

The serotonergic system undergoes adaptive changes after, but not before, MC loss [96]. Degeneration of MCs causes a decrease in serotonergic input received by the OB, whereas the number of serotonergic cells in the raphe nuclei remains constant. In this regard, the neurotrophin BDNF and its main receptor TrkB exhibit altered expression in the OBs of *pcd* animals even before the loss of MCs [96].

Although the expression of noradrenaline transporters is not affected by MC degeneration, variations in adrenergic receptors in some olfactory regions were defined, suggesting a local regulation of the NA system in regions influenced by MC loss [82]. The *pcd* mice also show reorganisation of zincergic centrifugal projections from the anterior olfactory nucleus to the OB, indicating that plasticity occurs in response to MC loss [97].

#### 5.2.3. Neural Plasticity in the Olfactory Bulb after Mitral Cell Loss

Neural progenitor cells from the rostral migratory stream differentiate into bulbar interneurons that modulate MC activity. Interestingly, changes in the proliferation rate, tangential and radial migration patterns and survival of newly generated neurons in *pcd* mice were reported. Consequently, the absence of MCs in these mutants elicits differences in the final destination of the newly generated interneurons. Moreover, the depletion of MCs also alters the survival of the newly generated interneurons, in accordance with the decrease in the number of synaptic targets available [98].

#### 5.2.4. Alterations in Olfactory Task Performance after Mitral Cell Loss

Despite the importance of the olfactory system in learning and affective behaviour in mice [99], little information about the potentially deleterious consequences of MC degeneration on olfactory-related task performance in *pcd* mice is available. Using precision olfactometry, Diaz and colleagues showed that after MC death, *pcd* mutants exhibit poor odourant detection ability and limited odour discrimination ability [100]. In particular, *pcd* mice are able to detect elevated, but not low, concentrations of odourants and discriminate them in a crude manner, suggesting the involvement of MCs in fine odour transmission and processing [100,101].

### 5.3. Degeneration in the Thalamus

Discrete populations of thalamic neurons degenerate in *pcd* mice between P50 and P60 and are nearly absent at P90 [21,102]. Thus, massive neuronal degeneration is observed in the central division of the mediodorsal nucleus, the ventral medial geniculate, posterior ventromedial and submedial nuclei, as well as portions of the ventrolateral and posteromedial nuclei that immediately surround the medial division of the ventrobasal complex. Degenerating thalamic neurons in the ventral medial geniculate nucleus, the main auditory thalamic area, show degenerative cellular hallmarks that resemble those reported for mutant PCs and MCs [102,103].

The electrophysiological and molecular changes in the ventral medial geniculate nucleus in *pcd* mice were also examined [103]. Likewise, a progressive decrease in auditory evoked potentials and NMDA receptor-dependent fast oscillations in the auditory cortex were detected in *pcd* mice [103].

Changes in the regional thalamic distribution of noradrenaline uptake sites, as well as in the expression of adrenergic receptors, were described following thalamic neuron loss in *pcd* mice [82]. In addition, increased levels of dopamine receptors were found in the centromedian thalamic nucleus in *pcd* mice [83].

### 5.4. Degeneration in the Retina

The onset of retinal degeneration of photoreceptors in *pcd* mice occurs between 3 and 5 weeks of age, when approximately 50% of receptors are quickly lost [104,105]. Afterward, degeneration progresses quite slowly, with approximately 10% of the photoreceptors remaining by one year of age [104,105], and rods degenerating faster than cones [105]. The main photoreceptor alterations include abnormal accumulation of “bead-like” vesicles and ribosomes, disruption of the Golgi apparatus, and a significant reduction in the number of connecting cilia [106], which ultimately lead to the death of photoreceptors by apoptosis [6,40,107]. In addition, the pcd mutation in photoreceptors increases their vulnerability to the cellular stress produced by constant light exposure [108]. Progressive accumulation of polyglutamylated tubulin was detected in parallel with the degeneration of *pcd* mutant photoreceptors [106]. Progressive loss of dendrites and disorganisation of axon terminals in retinal bipolar cells were also reported in parallel with degeneration of photoreceptors [41].

Consistent with cellular alterations, electroretinography of the *pcd* mutant retina has revealed a progressive reduction in the amplitude of electrical signals in both rods and cones at advanced stages of degeneration in comparison with that in the control retina [41].

### 5.5. Degeneration of Other Neuronal Types

Quantitative estimation of the number of α-motor neurons in the ventral horn of the lumbar spinal cord has revealed an approximately 50% reduction in the number of these cells in *pcd* mice compared with control mice, which is accompanied by dysregulation of tubulin polyglutamylation [1]. Moreover, peripheral nerve degeneration with reduced motor nerve caliber, significant loss of myelinated axons and altered axon morphology were reported in *pcd* mice [1].

As mentioned, one of the main pathological hallmarks in CONDCA patients is muscle weakness. Additionally, AGTPBP1 mRNA was found to be expressed in mouse skeletal muscle [12]. Muscle tissue organisation in *pcd* mice was found to be hardly affected, with no obvious accumulation of collagen or fibrosis. However, the diameter of skeletal myofibres is reduced compared with that of control myofibres, most likely due to ataxia-derived atrophy (Figure 3E,F) [37] resulting from the decrease in the number of α-motor neuron axons innervating the skeletal muscle [1]. As in other tissues affected by AGTPBP1 loss of function, whole-protein extracts from *pcd* skeletal muscle exhibit higher levels of tubulin polyglutamylation than those from control skeletal muscle [15].

### 5.6. Therapeutic Strategies

Due to AGTPBP1 mutation-mediated primary neuronal death and the occurrence of secondary neurodegenerative processes in the *pcd* mouse brain, this mouse model may serve as an attractive model for investigating new neuroprotective strategies to prevent, or at least attenuate, neurodegeneration. Most of the experimental therapies assessed in the *pcd* mice presented here aimed to reverse cerebellar degeneration in *pcd* mutants. Stem cell-based neuroregeneration or the use of molecules with neuroprotective potential are the main experimental approaches that were assessed in *pcd* mice.

#### 5.6.1. Stem Cell-Based Transplantation

Under neurodegenerative conditions, grafted cells from healthy donors may provide neurotransmitters with neuroprotective potential, replace degenerated neurons and provide trophic support to surviving neurons. Based on this notion, embryonic cerebellar grafts appear to be a potential therapeutic strategy not only to replace PCs but also to prevent secondary neuronal death. Cells from solid embryonic cerebellar grafts from healthy donors implanted into 3-month-old *pcd* mutants were able to migrate, settle and establish functional synapses in the host cerebellar cortex [109,110,111,112,113]. Similarly, suspended normal embryonic cerebellar cells transplanted into the *pcd* mouse cerebellum were shown to survive and integrate with the degenerative harmful host environment, develop the characteristic PC cytoarchitecture and re-establish host-to-graft afferent innervation, while also ameliorating motor deficits [114,115,116,117,118,119].

Bone marrow-derived stem cell (BMDSC) transplantation also appears to be a therapeutic option for ameliorating neurodegeneration in *pcd* mice. Initial studies have indicated that grafted BMDSCs in *pcd* mice are able to migrate and reach the degenerating cerebellum and OB, although most of them differentiate into glial cells [120]. Posterior bone marrow transplantation notably improves skeletal muscle tissue organisation rather than attenuating neurodegeneration, which correlates with a partial but significant restoration of locomotor performance [37]. Thus, recovery of muscular dysfunction appears to be the basis of this locomotor improvement. BMDSC transplantation in *pcd* mice also results in the attenuation of MC degeneration and an associated improvement in odour detection [100]. Limitations of this approach include that the delivery of healthy BMDSCs to the damaged site is not fast enough to stop neuronal loss over time. Thus, optimisation of this technique by ensuring a regular supply of healthy stem cells through continuous, daily transplants would increase the population of pluripotent cells that reach the target tissue and potentially fuse with unaffected mutant PCs, increasing their survival [121].

An additional limitation is the physical barrier of the granule cell layer, which impedes healthy grafted cells from reaching the PC layer. In addition, the complexity of cerebellar circuitry is too great for it to be finely reconstructed. Moreover, the purposed fate of grafted cells is strictly regulated by a large variety of factors that may vary according to each degenerative environment. Therefore, neurotransplantation and stem cell-based therapy in patients with cerebellar degeneration are still far from being practical [122].

#### 5.6.2. Preservation of Degenerating Neurons in *pcd* Mice

Other experimental approaches have aimed to protect and preserve mutant PCs through the administration of neuroprotective molecules, the exogenous administration of functional AGTPBP1 and the directed modulation of specific signalling pathways involved in the degeneration of PCs in *pcd* mice.

The neuroprotective role of insulin-like growth factor (IGF-I) was shown, and IGF-I has been extensively used to treat several neurodegenerative disorders. Interestingly, reduced levels of IGF-I were found in patients with cerebellar dysfunction and ataxia [123]. Consistently, IGF-1 administration in *pcd* mice was shown to significantly increase body weight and survival and improve motor performance [124]. Interestingly, administration of IGF-1 in patients with autosomal dominant cerebellar ataxia delays the progression of the disease and appears to be a potentially promising therapeutic option for CONDCA patients [125]. In addition, oleoylethanolamide, an endocannabinoid compound, was proposed to prevent neuronal damage, delay PC death and ameliorate cognitive decline in *pcd* mice [126].

Affectation in the auditory cortex in *pcd* mice following thalamic neuron degeneration is closely related to a marked upregulation of NMDA expression. Accordingly, the administration of an NMDA antagonist restores the electrophysiological response evoked in the auditory cortex in *pcd* mice [103].

Another important target of neuroprotective agents is the modulation of the glial response. Accordingly, a harmful glial response in the cerebella of *pcd* mice could be directly related to the rapid degeneration of PCs [46]. In this regard, attenuation of glial activation following minocycline administration delays the death of PCs in *pcd* mice and mildly improves their locomotor performance [52]. Thus, administration of glial activation inhibitors or genetic modulation of the glial response may be considered potential therapeutic approaches to ameliorate neurodegenerative disorders [127].

#### 5.6.3. Genetically Mediated Therapeutic Approaches

Genetically mediated restoration of functional AGTPBP1 expression in both the cerebellum and the retina is sufficient to rescue PC and photoreceptor degeneration in *pcd* mice [14,30]. As mentioned above, AGTPBP1 loss of function leads to excessive tubulin polyglutamylation, which seems to be the main cause of neurodegeneration in *pcd* mice. Based on this notion, the abnormal accumulation of polyglutamylation in the mutant cerebellum may be rescued by inactivation of the polyglutamylase tubulin-tyrosine ligase-like 1 (TTL1), resulting in almost complete preservation of PCs in the *pcd* mouse cerebellum [22]. This raises the possibility that pharmacologically mediated regulation of enzymes counteracting AGTPBP1-mediated reactions, such as TTL1, could be considered a new therapeutic approach for the treatment of AGTPBP1-related diseases.

## 6. Conclusions

Here, we summarise the most recent experimental findings that support *AGTPBP1* as the gene responsible for the development of CONDCA in humans. Additionally, the pathogenic events that occur in *pcd* mice, which harbours loss-of-function mutations in the *AGTPBP*1 gene, are described in-depth and summarised in the graphical abstract. The fact that the pathological characteristics of the pcd mutation share clear similarities with those of CONDCA patients indicates that AGTPBP1 function is essential to the development of neurological disorders not only in mice but also in humans. In this regard, it is evident that the *pcd* mouse appears as a promising model for the development of new therapeutic strategies for clinical trials in humans. Polyglutamylation inhibition was recently described as a promising therapeutic option for CONDCA patients. This line of investigation together with others involving the use of *pcd* mice and those included in this review may be considered in the future as therapeutic options for CONDCA treatment.

## Figures and Tables

**Table 1:** Identified biallelic variants in the *AGTPBP1* gene. M: Male F: Female. * means substitution.

Patient (Age; Sex; Consanguinity)	Allelic Variant	Consequence	Region Affected	Reference
2-year-old; F; NO	NM_001330701 c.2336-1G>T	Transversion in intron 17. Results in a splice site aberration, a frameshift and premature termination (M780fs)	Cytosolic carboxypeptidase N-terminal domain	[1]
	NM_001330701 c.2736delC	Deletion in exon 21. Results in a frameshift and premature termination (T912Ter)	Zinc-carboxypeptidase domain	[1]
12-month-old; M; YES	NM_001330701 c.2752C>T	Transition in exon 21. Results in R918W substitution	Zinc-carboxypeptidase domain	[1]
7-month-old; M; YES	-	Deletion of exons 1 to 12	Non-defined	[1]
Not available	NM_015239.2 c.2632C>T	Cerebellar hypoplasia and lower motor neuron degeneration. Results in R878W substitution	Zinc-carboxypeptidase domain	[4]
4-year-old; M; NO	NM_001286715 c.2351A>G	Results in a T784C substitution	Non-defined	[2]
	NM_001286715 c.2998C>T	Results in a frameshift and premature termination (R1000Ter)	Zinc-carboxypeptidase domain	
15-month-old; M; YES	NM_001286715 c.2342C>T+2T>G	Skips exon 15 (loss of 29 highly conserved aa)	Non-defined modular domain	[2]
5-year-old; F; NO	NM_001330701 c.2752C>T	Transition in exon 21. Results in R918W substitution	Zinc-carboxypeptidase domain	[1]
	NM_001330701 c.2080T>G	Transversion in exon 15. Results in a Y694D substitution	Non-defined	
16-month-old; F; YES	NM_001330701 c.2566C>T	Homozygous transition in exon 19. Results in a Q856 *	Non-defined	[1]
8-year-old; M; YES	NM_001330701 c.2395C>T	Results in a R799C substitution	Cytosolic carboxypeptidase N-terminal domain	[1]
8-year-old; M; YES	NM_001330701 c.2566C>T	Results in a P799C substitution	Cytosolic carboxypeptidase N-terminal domain	[1]
7-month-old; M; YES	NM_001330701 c.2396G>T	Results in a P799L substitution	Cytosolic carboxypeptidase N-terminal domain	[3]
2-year-old; M; YES	NM_001330701 c.2396G>T	Results in a P799L substitution	Cytosolic carboxypeptidase N-terminal domain	[3]
20-month-old; F; NO	NM_001330701 c.988C>T	Results in a R330 *	Non-defined	[1]
		Results in a Y912X substitution	Zinc-carboxypeptidase domain	
8-year-old; M; YES and 5-year-old; F; YES	NM_001330701 c.2728C>T	Results in a R910C substitution	Zinc-carboxypeptidase domain	[1]
3 infant sibs; YES	NM_001330701 c.2362C>T	Transition in exon 18. Results in a Q788 *	Cytosolic carboxypeptidase N-terminal domain	[1]
14-year-old; M; NO	NM_001330701 c.2552C>T	Transition in exon 19, resulting in a T851M substitution	Non-defined	[1]
	NM_001330701 c.2969A>T	transversion in exon 22, resulting in a H990L substitution	Zinc-carboxypeptidase domain	
21-month-old; M; YES	NM_001330701 c.3293G>A	Mutation in exon 24, resulting in a S1098N substitution	3′ end domain	[36]
17-year-old; F; YES	NM_001330701 c.3293G>A	Mutation in exon 24, resulting in an S1098N substitution	3′ end domain	[36]

**Table 2:** Clinical findings reported for patients harboring *AGTPBP1*-mutated gene.

Feature	Data from [1,2,3,4,36]
Onset	Birth to 20 months
Gender	10F, 9M Not available (1)
Consanguinity	14/19 Not available (1)
Progressive degenerative course	19/20
Microcephaly	11/20 Not available (1)
Motor delay	20/20
Hypotonia	19/20 Not available (1)
Muscle weakness	16/18 Not available (2)
Muscle weakness pattern	Tetraparesis/plegia (8) Lower limb (2) Neck (3) Diaphragm/intercostal (4) Not specified (8)
Muscle atrophy	9/18
Tongue fasciculations	7/20 Not available (13/20)
Tendon reflexes	Low or absent (10/20) Normal (3/20) Increased (6/29) Not available (1)
Ataxia	Yes (6) Not available (12)
Dystonia	5/20 Not available (1)
Spasticity	7/20 Not available (3/18)
Respiratory distress	9/20 Not available (1)
Feeding difficulties	13/20 Not available (1)
Eye movement abnormalities	Detected (12/20) Not detected (6/20) Not available (2/20)
Hearing	Impaired (1/20) Normal (5/20) Not available (14/20)
Cognitive delay	17/20 Not available (3/20)
Brain MRI	Cerebellar atrophy (18/20) Dysplastic corpus callosum (6/20) Small pons (1/20) Enlarged CSF spaces (1/20)
Nerve conduction studies	Motor neuropathy (2/20) Axonal motor neuropathy (5/20) Normal (1/18) Not available (12/20)
Electromyography	Denervation (5/20) Neurogenic (2/20) Normal (1/20) Not available (12/20)

**Table 3:** Summary of AGTPBP1 reported mutant alleles. ENU: N-ethyl-N-nitrosourea.

Allele Name Mutation	Mutation	Clinical Features	Genetic Mutation in *AGTPBP1*
Agtpbp1^pcd−1J^	Spontaneous	Reduced body size; Ataxia; cerebellar atrophy; postnatal degeneration of thalamic neurons, PCs, MCs and retinal photoreceptors; male infertility; female partial fertility.	Unknown (possibly in regulatory region)
Agtpbp1^pcd−2J^	Spontaneous	Hylomorphic allele with reduced	Insertion (~7.8Kb) between exons 14–15
Agtpbp1^pcd−3J^	Spontaneous	Reduced body size; Ataxia; Cerebellar atrophy; postnatal degeneration of thalamic neurons, PCs, MCs and photoreceptors; male infertility; female partial fertility; Reduced number of antral follicles.	Deletion (~12.2 Kb) between intron 5 and exon 8
Agtpbp1^pcd−4J^	ENU-induced mutagenesis	Ataxia; degeneration of PCs	Unknown
Agtpbp1^pcd−5J^	Spontaneous	Ataxia; Degeneration of PCs and MCs	Insertion of an aspartic acid residue (D775) in exon 18
Agtpbp1^pcd−6J^	ENU-induced mutagenesis	Ataxia; cerebellar and testicular atrophy; postnatal degeneration of PCs, MCs and photoreceptors; decreased skeletal muscle fiber size; male infertility.	Unknown
Agtpbp1^pcd−7J^	Spontaneous	Ataxia; postnatal degeneration of PCs; enlarged hippocampus; abnormal hearing	Unknown
Agtpbp1^pcd−8J^	Spontaneous	Affectation of behavior; low size body; Alteration of nervous system development, reproductive, and vision.	Unknown
Agtpbp1^pcd−9J^	Spontaneous	Ataxia, but has a slightly later onset than that caused by the original *pcd* allele.	Unknown
Agtpbp1^pcd-Tg(Dhfr)1jwg^	Transgene insertion	Ataxia; degeneration of PCs, MCs and photoreceptor cells; some male infertility, female partial fertility; degeneration of sperm	Random gene disruption
Agtpbp1^Drunk^	Mutagenesis	Degeneration of Purkinje cells and photoreceptor cells; Male infertility	Unknown
Agtpbp1^Rio^	Mutagenesis	Tremor and abnormal sperm	Unknown
Agtpbp1^babe^	ENU-induced mutagenesis	Ataxia; paraparesis	P804 arginine to a termination codon
Agtpbp1^pcd-Btlr^	ENU-induced mutagenesis	Ataxia; degeneration of PCs, MCs and photoreceptor cells; male infertility, oligozoospermia and teratozoospermia	a T-to-A transversion in the donor splice site of intron 11
Agtpbp1^pcd-m2Btlr^	ENU-induced mutagenesis	Tremors; decreased body size; reduced activated sperm motility	an A to G transition; destroys the acceptor splice site of intron 7 of the gene
Agtpbp1^pcd-Sid^	Spontaneous	Reduced body size; Ataxia; Cerebellar atrophy.	Deletion of exon 7
Agtpbp1^Gt(IST13517F11)Tigm^	Gene trapped allele	one ES cell; unclassified	Chr13:59477801-59478055 bp (-);Chr13:59477801-59477979 bp (-)
Agtpbp1^Gt(OST186151)Lex^	Gene trapped allele	Lex-1 (ES Cell)	Chr13:59531904-59544452 bp (-)
Agtpbp1^Gt(OST188387)Lex^	Gene trapped allele	Lex-1 (ES Cell)	Chr13:59531902-59533237 bp (-)
Agtpbp1^Gt(OST252171)Lex^	Gene trapped allele	Lex-1 (ES Cell)	Chr13:59531904-59544452 bp (-)
Agtpbp1^Gt(OST300426)Lex^	Gene trapped allele	Lex-1 (ES Cell)	Chr13:59531904-59544452 bp (-)
Agtpbp1^Gt(OST300428)Lex^	Gene trapped allele	Lex-1 (ES Cell)	Chr13:59536248-59536374 bp (-)
Agtpbp1^Gt(OST301743)Lex^	Gene trapped allele	Lex-1 (ES Cell)	Chr13:59531913-59536374 bp (-)
*pcd* ^KO^	Knock-out	Ataxia; cerebellar atrophy, postnatal degeneration of PCs and photoreceptors.	Deletion of exons 21 and 22

**Table 4:** Comparison of pathological findings between CONDCA patients and the pcd mutant mouse. N.E: Not examined.

Physiopathological Feature	CONDCA Patients	*pcd* Mice
Early-onset	YES	YES
Progressive degenerative course	YES	YES
Microcephaly	YES	YES
Motor delay	YES	YES
Hypotonia	YES	N.E
Muscle weakness	YES	YES
Muscle atrophy	YES	YES
Tongue fasciculations	Frequent	N.E
Alteration of tendon reflexes	Frequent	N.E
Ataxia	Frequent	YES
Dystonia	Frequent	N.E
Spasticity	Frequent	N.E
Respiratory distress	Frequent	N.E
Feeding difficulties	Frequent	YES
Eye movement abnormalities	Frequent	N.E
Defective hearing	Occasional	YES
Cognitive delay	YES	YES
Motor and axonal motor neuropathy	Frequent	YES
Denervation	Frequent	YES
Olfactory dysfunction	N.E	YES
Visual deficiency	N.E	YES
Defective sperm	N.E	YES
